# Prediction of Response in Melanoma Therapy by Systemic Inflammatory Response – One Size Fits Not All

**DOI:** 10.1016/j.ebiom.2017.03.032

**Published:** 2017-03-24

**Authors:** Georg Richtig, Martin Pichler

**Affiliations:** a Institute of Experimental and Clinical Pharmacology, Medical University of Graz, Graz, Austria; b Department of Dermatology, Medical University of Graz, Graz, Austria; c Division of Oncology, Department of Internal Medicine, Medical University Graz, Graz, Austria; d Department of Experimental Therapeutics, The University of Texas MD Anderson Cancer Center, Houston, TX, USA

Immunotherapy in melanoma had a long history and tradition. Early efforts have been made with interferon-alpha and interleukin-2 in melanoma patients with the main focus to augment the function of immune cells to fight this deadliest type of skin cancer. Although a huge amount of experimental data and many case reports or case series demonstrated that melanoma is a very immunogenic tumor, the clinical benefit of early immunotherapeutic approaches with those agents was limited and came with the price of severe and frequent adverse events. (Lesterhuis et al., 2011) The real immunotherapy breakthrough came with the discovery and characterization of immune checkpoint molecules such as CTLA-4 and the consequent development and clinical testing of a monoclonal antibody directed against CTLA-4 (i.e., ipilimumab or IPI). (Sharma and Allison, 2015) This new drug stands for a revolution in cancer medicine and can now be considered as first line therapy in *BRAF*^*wt*^ as well as in *BRAF*^*mt*^ metastatic melanoma. (Richtig et al., 2017) It was approved for melanoma treatment based on the significantly improved overall survival and higher response rate compared to the actual standard of care chemotherapeutic drug. (Hodi et al., 2010) On the one hand IPI stands for a new era in cancer care with unbelievable so called long term responders, as some patients experience an unpredictable stabilization and even complete remission of their disease. (Schadendorf et al., 2015) On the other hand, these encouraging results drove the development of several other immune checkpoint inhibitors including monoclonal antibodies against PD-1, PD-L1, Tim-3, Lag-3, OX40 and others. Some of them, especially PD-1 and PD-L1 have now been approved for many other types of cancer. (Sharma and Allison, 2015) However, several problems are accompanied with the use of IPI in melanoma patients: Firstly, objective response rate upon administration of IPI treatment is rather low with ~ 11%; Secondly, financial and personal costs are enormous; Thirdly, many patients experience severe grade IV adverse effects. To support clinicians in their decision making process, biomarkers for predicting which patient will benefit and which one will derive harm are urgently needed to better predict clinical outcome variables like objective response, progression free survival, long term survival or occurrence of immune-related adverse events. This question has been addressed by several groups and laboratory parameters, epigenetic parameters and others have been proposed as biomarker in patients treated with IPI (Smolle et al., 2017).

Markers of the systemic inflammatory response including C-reactive protein, neutrophil-lymphocyte ratio or others have been proposed as prognostic and predictive biomarkers in many types of cancer (Stotz et al., 2013, Szkandera et al., 2014).

In this *EBioMedicine* study, Cassidy et al. investigated the prognostic value of the Neutrophil to Lymphocyte ratio (NLR) in patients with stage IV melanoma receiving monotherapy with either IPI (3 mg kg^− 1^ or 10 mg kg^− 1^) or BRAF inhibitors (BRAFi) (vemurafenib 960 mg or dabrafenib 150 mg). (Cassidy et al., 2017) The novelty of this study includes that NLR was calculated sequentially for four times (pretreatment baseline and every three weeks for three times until week nine). The NLR values were stratified into NLR low < 5 and NLR high ≥ 5. The study group could show that in IPI treated patients NLR ≥ 5 was significantly associated with worse overall survival, progression free survival and lower objective response rates and this result was successfully validated by multivariate analysis.

Although it was known that high pretreatment NLR is accompanied with disease specific survival in IPI-treated patients, Cassidy et al. could show that NLR can be assessed within any time point of treatment. (Cassidy et al., 2017) This has important clinical implications for two reasons: Firstly, NLR is an easily measureable and broadly available routine parameter which can probably predict patients relapse before imaging. Secondly, IPI has been successfully tested as an adjuvant therapy in stage III melanoma were NLR could probably serve as biomarker for response as well as for a potential relapse, though this hypothesis has to be tested in a prospective manner. (Eggermont et al., 2016) The implication for current clinical scenario might be that when physicians see patients who develop high NLR during IPI treatment, one should think about earlier imaging and shorter follow-up appointments to earlier identify disease progression. However, though this study addresses very important questions and brings interesting insights, further research is necessary to provide us with more data on biomarkers and multiple biomarker approaches. One limitation of this study is that the development of immune checkpoint inhibitors led to the introduction of PD-1 inhibitors which currently represents the standard of care in melanoma patients. Thus, future prospective studies should address the same important hypothesis in patients treated with this new generation of immunotherapeutic agents.

## Conflicts of Interest

The authors declared no conflicts of interest.

GR received funding from the Austrian Science Fund FWF (W1241) and the Medical University Graz through the PhD Program Molecular Fundamentals of Inflammation (DK-MOLIN).
